# Outcomes Associated With Social Distancing Policies in St Louis, Missouri, During the Early Phase of the COVID-19 Pandemic

**DOI:** 10.1001/jamanetworkopen.2021.23374

**Published:** 2021-09-01

**Authors:** Elvin H. Geng, Joshua Schwab, Randi Foraker, Branson Fox, Christine M. Hoehner, Mario Schootman, Aaloke Mody, William Powderly, Byron Yount, Keith Woeltje, Maya Petersen

**Affiliations:** 1 Division of Infectious Diseases, Department of Medicine, Washington University in St Louis, St Louis, Missouri; 2 Institute for Public Health, Washington University in St Louis; 3 Division Biostatistics, School of Public Health, University of California, Berkely; 4 Division of General Internal Medicine, Department of Medicine, Washington University in St Louis, St Louis, Missouri; 5 BJC HealthCare, St Louis, Missouri; 6 SSM Health Saint Louis University Hospital, St Louis, Missouri; 7 Mercy Health, St Louis, Missouri; 8 Division of Biostatistics, School of Public Health, University of California at Berkeley, Berkeley

## Abstract

**Importance:**

In the absence of a national strategy in response to the COVID-19 pandemic, many public health decisions fell to local elected officials and agencies. Outcomes of such policies depend on a complex combination of local epidemic conditions and demographic features as well as the intensity and timing of such policies and are therefore unclear.

**Objective:**

To use a decision analytical model of the COVID-19 epidemic to investigate potential outcomes if actual policies enacted in March 2020 (during the first wave of the epidemic) in the St Louis region of Missouri had been delayed.

**Design, Setting, and Participants:**

A previously developed, publicly available, open-source modeling platform (Local Epidemic Modeling for Management & Action, version 2.1) designed to enable localized COVID-19 epidemic projections was used. The compartmental epidemic model is programmed in R and Stan, uses bayesian inference, and accepts user-supplied demographic, epidemiologic, and policy inputs. Hospital census data for 1.3 million people from St Louis City and County from March 14, 2020, through July 15, 2020, were used to calibrate the model.

**Exposures:**

Hypothetical delays in actual social distancing policies (which began on March 13, 2020) by 1, 2, or 4 weeks. Sensitivity analyses were conducted that explored plausible spontaneous behavior change in the absence of social distancing policies.

**Main Outcomes and Measures:**

Hospitalizations and deaths.

**Results:**

A model of 1.3 million residents of the greater St Louis, Missouri, area found an initial reproductive number (indicating transmissibility of an infectious agent) of 3.9 (95% credible interval [CrI], 3.1-4.5) in the St Louis region before March 15, 2020, which fell to 0.93 (95% CrI, 0.88-0.98) after social distancing policies were implemented between March 15 and March 21, 2020. By June 15, a 1-week delay in policies would have increased cumulative hospitalizations from an observed actual number of 2246 hospitalizations to 8005 hospitalizations (75% CrI: 3973-15 236 hospitalizations) and increased deaths from an observed actual number of 482 deaths to a projected 1304 deaths (75% CrI, 656-2428 deaths). By June 15, a 2-week delay would have yielded 3292 deaths (75% CrI, 2104-4905 deaths)—an additional 2810 deaths or a 583% increase beyond what was actually observed. Sensitivity analyses incorporating a range of spontaneous behavior changes did not avert severe epidemic projections.

**Conclusions and Relevance:**

The results of this decision analytical model study suggest that, in the St Louis region, timely social distancing policies were associated with improved population health outcomes, and small delays may likely have led to a COVID-19 epidemic similar to the most heavily affected areas in the US. These findings indicate that an open-source modeling platform designed to accept user-supplied local and regional data may provide projections tailored to, and more relevant for, local settings.

## Key Points

**Question:**

Given the geographic heterogeneity of the COVID-19 pandemic, is it possible to assess the outcomes of delayed social distancing policies within any one geographic location?

**Findings:**

In this decision analytical model of 1.3 million people in St Louis, Missouri, a delay of 2 weeks in public health policies initiated on March 17, 2020, was estimated to be associated with a nearly 6-fold total increase in deaths due to COVID-19 by June 15, 2020.

**Meaning:**

These findings suggest that timely local social distancing policies are associated with the number of COVID-19–related hospitalizations and deaths; local public health policies may avoid more severe pandemic consequences even in a widespread pandemic.

## Teaser

This decision analytical model investigates the association of delays in public health policy on social distancing during the COVID-19 pandemic and the number of hospitalizations and deaths from the disease in St Louis, Missouri.

## Introduction

In the absence of vaccines or highly efficacious treatment, many communities in the US undertook social distancing—a reduction in person-to-person contact—to mitigate the initial spread of COVID-19 in the spring of 2020. Policies for social distancing sought to reduce spread through closure of schools, limiting traffic at businesses, prohibiting large gatherings, and asking residents to reduce nonessential travel outside the home (eg, shelter in place). In the St Louis region of Missouri, and in many similar places, these restrictions also incurred a profound cost: businesses lost revenue, people lost wages, and health systems curtailed normal activities. Beginning on March 13, 2020, St Louis City and County enacted a number of such policies to limit the size of gatherings and reduce business traffic, which culminated in a shelter-in-place order that went into effect March 23, 2020. St Louis did not experience the epidemic severity seen in places such as New York City; as a result, some have argued that such shelter-in-place policies were out of proportion to infection threat.

The effects of social distancing policies on the course of the COVID-19 epidemic in any given municipality are, however, not obvious from casual inspection of case load or death data. Locations that undertook social distancing policies by definition cannot observe what would have happened had no policies been enacted (or if they had been enacted later). Furthermore, because the severity of the COVID-19 epidemic in a locality such as St Louis is likely to differ, perhaps markedly, from a regional or national average, mathematical modeling studies that provide national, global, or statewide projections under different scenarios shed relatively little light on local policy effects (eg, in St Louis).^1^ Comparisons to areas without social distancing policies can provide insights; however, those places likely differ in ways (eg, in behavior and mobility) that make them a poor proxy for what would have happened, even with statistical adjustments. Nevertheless, as the current epidemic continues to wax and wane, and as we prepare for future pandemic crises, some quantification of the outcomes of past difficult policy decisions is needed to inform future actions.

Although the consequences of delays in policies are by definition unobserved, infectious pathogens such as SARS-CoV-2 do behave in quantifiable and somewhat predictable ways. Research has yielded probable values for the reproductive number, latent period, infectious duration, and other parameters that can be used to project the epidemic course.^2,3,4^ In this study, we used a publicly available modeling platform (the Local Epidemiological Modeling for Management and Action or LEMMA, version 2.1) that we previously designed to facilitate local epidemic projections for the COVID-19 pandemic to project what would have happened in St Louis had social distancing policies been delayed.^5^ The parameter inputs (eg, transmission parameters, population size, and date and outcomes of social distancing policies) and the data to which they were calibrated (eg, daily hospital census) can be readily specified by users in a particular location and thereby offer potentially more locally relevant projections. We used this platform to examine the potential outcomes of 1-, 2-, or 4-week delays in social distancing policies in St Louis. The analysis also represents a case study in the use of the LEMMA platform for conducting similar analyses in other localities.

## Methods

### Model Overview and Data Sources

**Figure 1.**
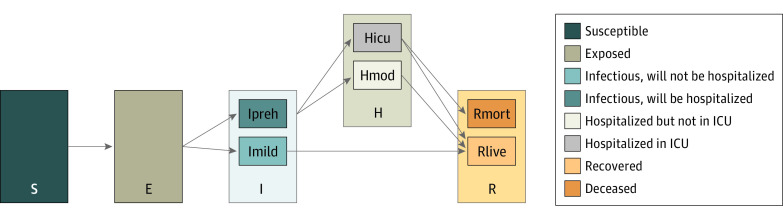
Compartmental Epidemiologic Model The Local Epidemic Modeling for Management & Action (LEMMA, version 2.1) model used in this analysis was a compartmental model of the COVID-19 epidemic with a susceptible-exposed-infectious-removed structure and several additional compartments. The infectious component was composed of 2 compartments for those who will be hospitalized (Ipreh) or will not be hospitalized (Imild). The hospitalized component was composed of 2 compartments representing admission to the intensive care unit (ICU) (Hicu) or no admission to the ICU (Hmod) within the hospital. Finally, the recovered component included a compartment for those who remained alive (Rlive) and those who were deceased (Rmort). E indicates exposed; I, infectious; R, removed; and S, susceptible.

The LEMMA model used in this analysis is a susceptible-exposed-infectious-removed compartmental epidemiologic model of the COVID-19 epidemic extended to integrate disease severity, hospitalization, use of the intensive care unit, and death (Figure 1). This model was created by several authors of this current study (E.H.G., J.S., and M.P.). The LEMMA model, Implemented in R, version 4.0.5 (R Foundation for Statistical Computing) and Stan, version 2.2.7 (NumFOCUS),^6^ was designed to meet the needs of local individuals working in public health agencies who are not mathematical modelers but who possess sophisticated locally relevant insights and data and who desire locally relevant models of the epidemic. Users can tailor the model output by supplying input parameters such as population size, as well as prior distributions on hospitalization rates (based on estimates of age strata–specific hospitalization rates^7^ standardized to local age structure), changing effective contact rate over time, and other values via an Excel (Microsoft Corp) interface. The model can then be calibrated using one or more sources of data from the region of interest (eg, hospital census, deaths, and new hospital admissions). The LEMMA model provides bayesian inference for parameter estimates and, under assumptions about future conditions, projections.

The study was approved by the Washington University Institutional Review Board, and a waiver for informed consent was granted because no identified data were used in the analysis. For this analysis, deidentified and aggregated hospitalization and death data for the St Louis region were drawn from the regional hospital systems of BJC HealthCare, SSM Health, and Mercy Health, which were housed by the Washington University Institute for Informatics. Data were used only from patients who resided within St Louis City and County. This study followed the Consolidated Health Economic Evaluation Reporting Standards (CHEERS) reporting guideline.

### Forecast Validation

We evaluated the accuracy of model projections for hospitalizations and deaths in St Louis County and City. A priori distributions for parameters including initial reproductive number, incubation period, latent period, and hospitalization rates within age strata were taken from the literature.^8,9,10^ To provide validation, forecast accuracy would ideally be assessed by calibrating the model on data only up to a given cutoff date using these priors and then comparing projected future hospitalizations and deaths with actual numbers observed but not used in calibration. However, such an approach is only expected to perform well for periods in which future effective contact rates (among other factors) remain constant; even a perfect model cannot infer from past data the timing and magnitude of future behavioral and policy changes. We therefore used a 2-step approach to assessing projection accuracy during the period of interest—a period in which policies and behaviors changed markedly over time, resulting in changes in reproductive number. In step 1, we calibrated the model using hospital census data through July 15, 2020, to estimate changes in effective contact rate (and effective reproductive number) over time. In step 2, we then recalibrated the model using data only through a given cutoff date (April 1, May 1, and June 1, 2020, respectively), using posterior distributions for the speed and magnitude of changes in effective contact rate (and therefore reproductive numbers) from step 1 as priors, but retaining our original prior distributions for all other parameters. We then compared projected hospitalizations and deaths over 12 weeks after the cutoff with observed numbers to assess the ability of the model to project the actual epidemic course. We provided median projections as well as the 25th and 75th percentiles.

### Statistical Analysis

To examine potential outcomes if social distancing policies were delayed, we used posterior distributions from the model calibrated on all hospitalization and death data through July 15, 2020, for all parameters other than the timing and magnitude of changes in effective contact rate. We then generated distributions of projected hospitalizations after March 20, 2020, under the hypothetical 1, 2, and 4-week delays in policy. Specifically, we projected outcomes if policies that in reality occurred on March 13 through March 23, 2020 (eFigure 1 in the Supplement) had instead occurred 1, 2, or 4 weeks later (but with unchanged effect sizes). For example, the 1-week delay scenario places the initial policy interventions on March 20 rather than March 13, and the shelter-in-place policy on April 1 rather than March 23. We then quantified the outcome of these delays by projecting hospitalizations and deaths over the 12 weeks after March 20 under each delay scenario. In the initial scenarios of delay, we assumed that social distancing behaviors would not have changed on their own without orders by the County or City (or other authorities). Although we found this plausible (given the absence of change in mobility in the weeks before March 13, irrespective of changes elsewhere in the country), we explored 2 sets of sensitivity analyses. First, we imposed a 25% reduction in reproductive number starting on March 13 to reflect the maximum plausible spontaneous behavior change that would have occurred in the short term had policy changes not been implemented. Second, we introduced an additional 50% reduction in reproductive numbers when hospital census hit a “panic” level (600, 800, or 1000 patients) that was near the initial hospital capacity for patients with COVID-19.

## Results

**Figure 2.**
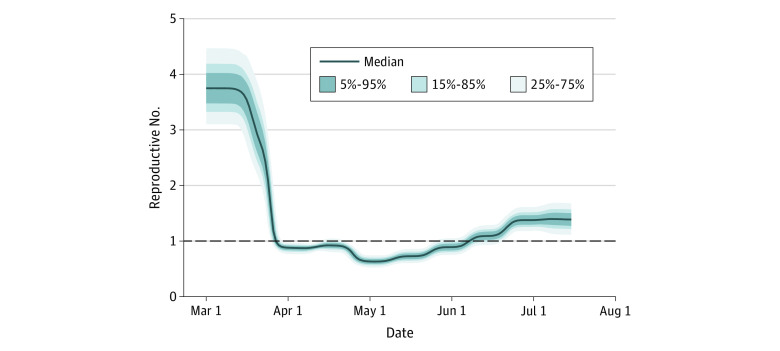
Estimated Effective Reproductive Number Over Time in St Louis City and County Output from modeling showing estimated reproductive number over time in the St Louis region based on hospitalization data. The blue line represents median projections and shaded bands represent the fraction of projections in the depicted interval. The black dashed line represents median projection of hospitalizations or deaths.

This study included data from 1.3 million people in St Louis, Missouri. We calibrated the model using daily hospital census data until July 31, 2020, and used population size as well as prior distributions on unknown parameters (eg, date and magnitude of changes in reproductive number, latent period) drawn from the literature. The full calibration used 23 473 total patient-hospital days of observations and yielded posterior distributions of unknown parameters that were the basis of projections of hospitalizations and deaths under counterfactual scenarios. We estimated an initial reproductive number in the St Louis region of 3.9 (95% credible interval [CrI], 3.1-4.5), which fell to 0.93 (95% CrI, 0.88-0.98) after social distancing policies were implemented between March 15 and March 21, 2020, a latent period of 2.2 days (95% CrI, 1.2-3.5 days), and a hospitalization rate of 3.9% (95% CrI, 1.2%-7%). We estimated that the effective reproductive number began to decrease on March 15 by 27% and decreased an additional 67% starting on March 21, with each change taking full effect over 7 days. Overall, the model suggested a slight increase in the reproductive number by 1.2-fold starting on April 5, with a smaller 20% decrease on April 19, resulting in a reproductive number of approximately 1 through April (Figure 2).

Using these estimates of reproductive number over time, we then supplied the model with actual hospitalization data only until April 1, May 1, and June 1 to examine concordance between actual and projected hospital census, cumulative admissions, and deaths for 12 weeks past the last date actual data were supplied (eFigure 2 in the Supplement). For example, 8 weeks (or 42 days) after April 1 (ie, on May 13), 422 deaths had occurred in actuality, whereas the model projected 432 deaths, a difference of 2.3%.

**Figure 3.**
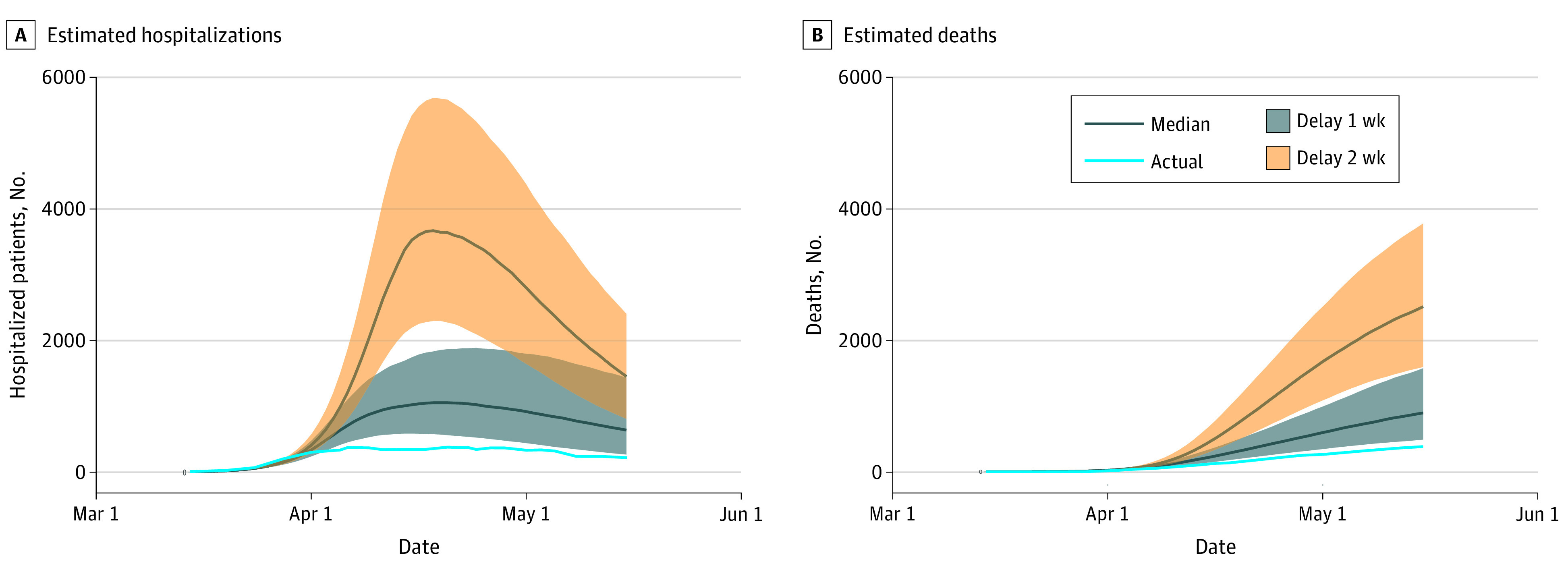
Hospitalizations and Deaths Associated With Delays in Social Distancing Policies Estimated hospitalizations (A) and deaths (B) with delays in social distancing policies. Shaded bands represent the 25th and 75th percentiles of projections.

Model projections that showed cumulative hospitalizations, hospital census data, and hospital deaths if the policies taken by St Louis City and County were delayed by 1 week demonstrated considerably greater epidemic severity (Table and Figure 3). With a 1-week delay, the estimated decreases in the reproductive number by 27% on March 15 and by 67% on March 21 would have instead occurred on March 22 and March 28, respectively. With such a 1-week delay, we projected that by April 15, cumulative hospitalizations would have risen from the 1011 observed to a median of 3020 (75% CrI, 1854-4885 hospitalizations), representing a 194% increase (75% CrI, 69%-400%). By May 15, the actual observed hospitalizations of 1865 would have risen to a median of 6584 (75% CrI, 3459-11967 hospitalizations), or a 253% increase (75% CrI, 86%-542%).

**Table.** Actual and Projected Hospital Census, Cumulative Hospitalizations, and Deaths Under a Number of Scenarios

Variable	Observed actual No.	Median (75% credible interval)					
		Scenario 1^a^	Scenario 2^b^	Scenario 3^c^	Scenario 4^d^	Scenario 5^e^	Scenario 6^f^
**Outcomes by April 15, 2020**							
Hospital census, No.	348	1025 (588 to 1745)	3527 (2197 to 5419)	6741 (4598 to 9621)	694 (413 to 1137)	2088 (1332 to 3260)	1980 (1296 to 2781)
Difference of hospital census, No.	NA	677 (240 to 1397)	3179 (1849 to 5071)	6393 (4250 to 9273)	346 (65 to 789)	1740 (984 to 2912)	1632 (948 to 2433)
Change in hospital census, %	NA	194.5 (69 to 401.4)	913.5 (531.3 to 1457.2)	1837.1 (1221.3 to 2664.7)	99.4 (18.7 to 226.7)	500 (282.8 to 836.8)	469 (272.4 to 699.1)
Cumulative admissions, No.	1011	3020 (1854 to 4885)	7992 (5155 to 12 253)	13 155 (8982 to 18 950)	2280 (1445 to 3509)	5221 (3425 to 7924)	5132 (3391 to 7375)
Difference of cumulative admissions, No.	NA	2009 (843 to 3874)	6981 (4144 to 11 242)	12 144 (7971 to 17 939)	1269 (434 to 2498)	4210 (2414 to 6913)	4121 (2380 to 6364)
Change in cumulative admissions, %	NA	198.8 (83.4 to 383.2)	690.5 (409.9 to 1112)	1201.2 (788.4 to 1774.4)	125.5 (42.9 to 247.1)	416.4 (238.8 to 683.8)	407.6 (235.4 to 629.5)
Cumulative deaths, No.	115	219 (141 to 337)	443 (293 to 676)	589 (400 to 871)	181 (120 to 266)	327 (220 to 479)	326 (218 to 466)
Difference in cumulative deaths, No.	NA	104 (26 to 222)	328 (178 to 561)	474 (285 to 756)	66 (5 to 151)	212 (105 to 364)	211 (103 to 351)
Change in cumulative deaths, %	NA	90.4 (22.6 to 193)	285.2 (154.8 to 487.8)	412.2 (247.8 to 657.4)	57.4 (4.3 to 131.3)	184.3 (91.3 to 316.5)	183.5 (89.6 to 305.2)
**Outcomes by May 15, 2020**							
Hospital census, No.	221	640 (269 to 1438)	1454 (809 to 2406)	3141 (1937 to 4707)	254 (120 to 539)	768 (428 to 1333)	406 (265 to 621)
Difference of hospital census, No.	NA	419 (48 to 1217)	1233 (588 to 2185)	2920 (1716 to 4486)	33 (–101 to 318)	547 (207 to 1112)	185 (44 to 400)
Change in hospital census, %	NA	189.6 (21.7 to 550.7)	557.9 (266.1 to 988.7)	1321.3 (776.5 to 2029.9)	14.9 (–45.7 to 143.9)	247.5 (93.7 to 503.2)	83.7 (19.9 to 181)
Cumulative admissions, No.	1865	6584 (3459 to 11 967)	18 079 (11 479 to 27 107)	42 157 (30 988 to 54 882)	3760 (2209 to 6400)	10 427 (6511 to 15 959)	7980 (5396 to 11 301)
Difference of cumulative admissions, No.	NA	4719 (1594 to 10 102)	16 214 (9614 to 25 242)	40 292 (29 123 to 53 017)	1895 (344 to 4535)	8562 (4646 to 14 094)	6115 (3531 to 9436)
Change in cumulative admissions, %	NA	253 (85.5 to 541.7)	869.4 (515.5 to 1353.5)	2160.4 (1561.6 to 2842.7)	101.6 (18.4 to 243.2)	459.1 (249.1 to 755.7)	327.9 (189.3 to 506)
Cumulative deaths, No.	384	898 (491 to 1578)	2517 (1598 to 3785)	5937 (4347 to 7675)	558 (330 to 910)	1485 (946 to 2251)	1215 (827 to 1715)
Difference in cumulative deaths, No.	NA	514 (107 to 1194)	2133 (1214 to 3401)	5553 (3963 to 7291)	174 (–54 to 526)	1101 (562 to 1867)	831 (443 to 1331)
Change in cumulative deaths, %	NA	133.9 (27.9 to 310.9)	555.5 (316.1 to 885.7)	1446.1 (1032 to 1898.7)	45.3 (–14.1 to 137)	286.7 (146.4 to 486.2)	216.4 (115.4 to 346.6)
**Outcomes by June 15, 2020**							
Hospital census, No.	99	199 (60 to 582)	233 (114 to 464)	350 (204 to 560)	53 (19 to 163)	124 (60 to 257)	50 (31 to 81)
Difference of hospital census, No.	NA	100 (–39 to 483)	134 (15 to 365)	251 (105 to 461)	–46 (–80 to 64)	25 (–39 to 158)	–49 (–68 to –18)
Change in hospital census, %	NA	101 (–39.4 to 487.9)	135.4 (15.2 to 368.7)	253.5 (106.1 to 465.7)	–46.5 (–80.8 to 64.6)	25.3 (–39.4 to 159.6)	–49.5 (–68.7 to –18.2)
Cumulative admissions, No.	2246	8005 (3973 to 15 236)	19 657 (12 484 to 29 290)	42 857 (31 368 to 55 811)	4124 (2353 to 7256)	11 164 (6972 to 17 204)	8092 (5489 to 11 487)
Difference of cumulative admissions, No.	NA	5759 (1727 to 12 990)	17 411 (10 238 to 27 044)	40 611 (29 122 to 53 565)	1878 (107 to 5010)	8918 (4726 to 14 958)	5846 (3243 to 9241)
Change in cumulative admissions, %	NA	256.4 (76.9 to 578.4)	775.2 (455.8 to 1204.1)	1808.1 (1296.6 to 2384.9)	83.6 (4.8 to 223.1)	397.1 (210.4 to 666)	260.3 (144.4 to 411.4)
Cumulative deaths, No.	482	1304 (658 to 2426)	3292 (2104 to 4905)	7331 (5390 to 9543)	695 (399 to 1199)	1904 (1181 to 2921)	1396 (952 to 1980)
Difference in cumulative deaths, No.	NA	822 (176 to 1944)	2810 (1622 to 4423)	6849 (4908 to 9061)	213 (–83 to 717)	1422 (699 to 2439)	914 (470 to 1498)
Change in cumulative deaths, %	NA	170.5 (36.5 to 403.3)	583 (336.5 to 917.6)	1421 (1018.3 to 1879.9)	44.2 (–17.2 to 148.8)	295 (145 to 506)	189.6 (97.5 to 310.8)
Peak hospital census, No. and date	381 (Apr 20)	1056 (Apr 19)	3670 (Apr 18)	10 377 (Apr 23)	700 (Apr 13)	2129 (Apr 18)	1980 (Apr 15)
Difference of peak hospital census, No.	NA	675	3289	9996	319	1748	1599
Difference of peak hospital census, %	NA	177.20	863.30	2623	83.70	458.80	419.70

Abbreviation: NA, not applicable.

^a^Delaying policy interventions by 1 week.

^b^Delaying policy interventions by 2 weeks.

^c^Delaying policy interventions by 4 weeks.

^d^Delaying policy interventions by 1 week but with 25% reduction in reproductive number due to spontaneous behavior change.

^e^Delaying policy interventions by 2 weeks but with 25% reduction in reproductive number due to spontaneous behavior change.

^f^Scenario 5 but with an additional 50% reduction in reproductive number when the hospitalization number reaches 800 patients.

We projected a similar worsening in deaths with a 1-week delay in policies. By April 15, a 1-week delay would have increased deaths from an actual observed of 115 to a median projected 219 (75% CrI, 141-337 deaths), representing a difference of 104 deaths (75% CrI, 26-222 deaths) and a 90% increase (75% CrI, 23%-193%). By May 15, the same 1-week delay would have increased deaths from the observed 384 actual deaths to a median projected 898 deaths (75% CrI, 491-1578 deaths), representing a difference of 514 deaths or a 134% increase. By June 15, a 1-week delay in policies was estimated to be associated with increased cumulative hospitalizations from an observed actual number of 2246 hospitalizations to 8005 hospitalizations (75% CrI, 3973-15 236) and increased deaths from an actual of 482 to a median 1304 projected (75% CrI, 658-2426 deaths), representing a difference of 822 (75% CrI, 176-7944 deaths) or a 171% increase (75% CrI, 37%-403%).

A 2-week policy delay, which implies changes in reproductive number of the same magnitude as observed, but occuring on March 29 and April 4 (instead of March 15 and March 21) yielded even greater epidemic severity. By May 15, we projected cumulative hospitalizations to change from the observed actual number of 1865 to a median projection of 18 079 (75% CrI, 11 479-27 107 hospitalizations) and deaths from an observed actual number of 384 to a median projection of 2517 (75% CrI, 1598-3785 deaths), representing an additional 2133 deaths (75% CrI, 1598-3785 deaths) or an increase of 555% (75% CrI, 316%-886%). By June 15, these figures would reach 19 657 cumulative hospitalizations and 3292 deaths (75% CrI, 2104-4905 deaths), corresponding to 2810 excess deaths associated with a 2-week delay, or a 583% increase.

We conducted sensitivity analyses (eTable in the Supplement) to assess the estimated outcome of a 2-week delay in policy accompanied by a 25% reduction in reproductive number—the largest reduction considered plausible—to account for spontaneous behavior change replacing social distancing policies in the short term (ie, occurring during the period of delay). In this analysis, by May 15, a median projected 1485 deaths (75% CrI, 946-2251 deaths) were estimated to occur instead of the actual observed 384 deaths, representing a difference of 1101 deaths (75% CrI, 562-1867 deaths) or an increase of 287% (75% CrI, 146%-486%). When we introduced a “panic” response to posit the occurrence of enough change in behavior to reduce the effective reproductive number by another 50% when hospitalizations reach a regional ceiling (using 800 as a conservative threshold), we found that by May 15, we would have seen a median 1215 deaths (75% CrI, 827-1715 deaths) instead of the observed 384 deaths.

## Discussion

In this decision analytical model study, we used an epidemiologic model of the COVID-19 epidemic tailored to the demographic and policy setting of St Louis, Missouri, and found that timely social distancing policies were associated with decreased numbers of hospitalizations and deaths. Even small, 1- to 2-week delays would likely have increased cumulative hospitalizations to levels that exceeded hospital capacity and resulted in approximately 3000 additional deaths. When taken in the context of the regional population of 1.3 million, we estimated that 2- to 4-week delays would have increased peak death rates from an actual rate of approximately 15 per 100 000 person-months to as much as nearly 100 per 100 000 person-months, which would have put St Louis on par with some of the worst affected regions in the US,^11^ such as Boston or New York. These projections are conservative in that we assumed that the mortality rate per hospitalization was unchanged across the total number of hospitalizations. In reality, it may be reasonable to assume that deaths per hospitalization would increase as hospitals neared or exceeded capacity. Results of a sensitivity analysis revealed that hypothetical spontaneous public behavior change in the absence of policies, including ones in which behavior changed markedly as hospitalizations reach capacity, do not avert severe scenarios. In 1917, during the influenza pandemic, St Louis implemented social distancing policies 3 weeks before Philadelphia and fared substantially better.^12^ In 2020, this modeling exercise suggests that St Louis again acted in a timely manner in the face of COVID-19.

Are these results credible? The answer depends in part on our perspectives on the plausibility of the hypothetical events in each of the alternative counterfactual scenarios. Would public behavior have changed rapidly in the absence of public health mandates? Many suggest that behavioral changes could or should occur without public policies but through “nudges.” Behavioral and implementation sciences have promoted theories about social cohesion, norms, zero-sum thinking, credibility, and communication, all of which represent means and methods to influence behavior.^13^ Although such perspectives are relevant and needed, the high reproductive numbers and rapid exponential growth seen during the early days of the COVID-19 epidemic in St Louis demanded one crucial ingredient for an effective public health response absent in most of these approaches: rapidity. Public health policies may have complex long-term effects and, in the current political climate, may trigger a backlash, but they can also exert immediate effects. Although it is not possible to know precisely what would have happened in the absence of a policy, rapid and drastic spontaneous behavior change would have been improbable within a week of actual policies, and unlikely within 2 weeks. Larger spontaneous change when hospitalizations reach a certain threshold are more probable, but our analyses suggest that by the time large numbers of people were hospitalized, even dramatic change would be too late to avoid a severe epidemic.

This study did not answer the question of whether or not strict social distancing guidelines resulted in acceptable outcomes, but our results suggest that lives saved vs revenue lost is perhaps a false dichotomy. Given the economic consequences of the pandemic—in particular, the fact that the African American community experienced a disproportionate burden of both the COVID-19 pandemic and the societal harms (ie, job loss) that have accompanied it—the trade-offs are by no means a zero-sum calculus.^14^ Approximately one-quarter of people in St Louis experienced wage or job loss (unpublished results). If social distancing policies had been implemented a few weeks later, it is doubtful that the economic costs would have been avoided, but the health benefits would have been markedly diminished. Instead, we need to create policies, such as paid sick leave for all and substantive income supplementation for those not covered by existing social safety nets, to ensure that most at-risk populations^15^ do not experience further negative outcomes as a cost of keeping society safe.^16^ Good public health policy is not necessarily at odds with, but rather supports, preservation of social function and livelihoods.

In addition to assessing the outcomes of policies in St Louis, this article provides a case study in the use of models such as LEMMA (ie, an open-source, publicly available, locally tailored model) as a means to explore location-specific potential outcomes of policies in many settings. Many regions do not have access to custom epidemiologic models developed for their locations. Local public health authorities in these areas could therefore make use of the LEMMA platform to repeat what has been done in this analysis. The LEMMA platform was designed with the ability to combine universal features of epidemics through use of a compartmental model with context-specific information (eg, hospitalizations from a particular location as well as locally informed transmission parameters). As vaccines for COVID-19 become more widely available but distribution strategies vary by location and new variants such as Delta emerge, regional and local projections remain important for public health situational awareness. The LEMMA model may also be widely useful (particularly the most recent version, which accommodates vaccine use and efficacy and variants) in much of the world where the supply of vaccine is unlikely to meet population needs in the near future.

### Strengths and Limitations

Use of a fairly simple underlying model structure is a strength of this study, given the limited amount of data available for calibration (particularly early in the epidemic) and the transparency and usability that such a structure provides to local policy makers. Furthermore, projections of hospitalizations under the estimated natural course of changes in effective contact rate were generally close to observed hospitalizations not otherwise used in generating projections; this finding supports, but does not guarantee, the model’s ability to capture the course of the epidemic over coming weeks.

This study had several limitations. First, the compartmental model encodes a number of simplifying assumptions, such as uniform mixing and a closed population, which do not fully reflect real-world behavior or conditions. Second, the plausibility of hypothetical delays in social distancing policies may be questioned. We used these hypothetical scenarios not because they would unequivocally have happened but to illustrate a potential scenario to contextualize the outcomes of decisions made in our region. Finally, we did not model disparities between African American and White populations given these delays, but we believe these differences would have been even greater than they were,^17^ which would be an unacceptable but probable outcome.

## Conclusions

The results of this decision analytical model study of the COVID-19 epidemic in St Louis, Missouri, suggest that short delays in social distancing policies could have incurred several thousand additional hospitalizations and deaths in the region. Public health mandates may not be a sustainable strategy for behavioral change, but they likely have a role when emergencies arise and rapid change is needed to ensure the safety of the public. Public health is often unrewarded precisely when most successful: averting a disaster tempts us to believe there was no disaster to avert. This study provides evidence for an opposite conclusion: in St Louis, timely public health actions may have helped the region avoid worse outcomes during the first wave of the COVID-19 pandemic.

## Notes

Supplement. **eMethods.** Overview of Compartmental Model **eFigure 1.** Date of Social Distancing Policies in St Louis **eFigure 2.** Projected Versus Actual COVID-19 Hospitalizations and Deaths **eTable.** Projected Hospitalizations and Deaths Under Additional Scenarios With Stronger Spontaneous Behavior Change
