# Supplementary material for: Outcomes Associated With Social Distancing Policies in St Louis, Missouri, During the Early Phase of the COVID-19 Pandemic
Source: JAMA Netw Open. 2021 Sep 1;4(9):e2123374. doi: 10.1001/jamanetworkopen.2021.23374 (PMC8411298; doi:10.1001/jamanetworkopen.2021.23374)
Supplement: Supplement. — eMethods. Overview of Compartmental Model eFigure 1. Date of Social Distancing Policies in St Louis eFigure 2. Projected Versus Actual COVID-19 Hospitalizations and Deaths eTable. Projected Hospitalizations and Deaths Under Additional Scenarios With Stronger Spontaneous Behavior Change [file jamanetwopen-e2123374-s001.pdf]

Supplementary Online Content

Geng EH, Schwab J, Foraker R, et al. Outcomes associated with social distancing policies in St Louis, Missouri, during the early phase of the COVID-19 pandemic. *JAMA Netw Open*. 2021;4(9):e2123374. doi:10.1001/jamanetworkopen.2021.23374

eMethods. Overview of Compartmental Model

eFigure 1. Date of Social Distancing Policies in St. Louis

eFigure 2. Projected Versus Actual COVID-19 Hospitalizations and Deaths

eTable. Projected Hospitalizations and Deaths Under Additional Scenarios With Stronger Spontaneous Behavior Change

This supplementary material has been provided by the authors to give readers additional information about their work.

© 2021 Geng EH et al. *JAMA Network Open.*

eMethods. Overview of Compartmental Model

The Local Epidemic Modeling for Management & Action (LEMMA) model used in this analysis is a discrete time compartmental epidemic model.[1] The version of the model (V 1.0; https://github.com/LocalEpi/LEMMA/tree/StLouis) used in the current analysis is an eight compartment model, which extends the classic four compartment Susceptible-Exposed-Infectious-Removed structure to incorporate disease severity, hospitalization and ICU use. Specifically, the population is conceptualized as existing in one of 8 states: 1) Susceptible to infection; 2) Exposed to SARS Cov-2, but not yet Infectious; 3a) Infected with SARS Cov-2 and Infectious to others with a more severe infection that will (with delay) lead to hospitalization; 3b) Infected with SARS Cov-2 and Infectious to others with a mild infection that will not lead to hospitalization; 4a) Hospitalized, but not in the ICU; 4b) Hospitalized in the ICU; 5a) Deceased; 5b) Recovered and Immune.

Model dynamics are deterministic on a discrete time scale. Specifically, on each day a fixed proportion of exposed become infected, a fixed proportion of infected become either hospitalized or recover and a fixed proportion of those in the ICU either die or recover. The proportion of those susceptible that become exposed is proportional to the number of infected and the time-varying effective contact rate parameter. Key epidemiologic and context parameters are treated as unknown and estimated based on local input data and user-specified prior distributions using Bayesian inference methods. Specifically, in the version of the model used in this analysis, posterior distributions on the following parameters were estimated: Basic reproductive number R0 before Intervention; Number of Days from Infection to Becoming Infectious (Latent Period); Duration of infectiousness (days); Time from onset of infectiousness to hospitalization (days); Average Hospital Length of Stay for Patients not in ICU (Days); Average Hospital Length of Stay for Patients in ICU (Days); Percent of Infected that are Hospitalized; Percent of Hospitalized COVID-19 Patients That are Currently in the ICU; Mortality Rate among ICU COVID-19 Patients. In the version of the model used in this analysis, the timing and magnitude of changes in the effective contact rate (and by extension, the effective reproductive number) constituted additional parameters with posterior distributions estimated during model calibration.

Point estimates and credibility intervals for unknown parameters are generated based on data inputs using the Stan programming language. Stan uses Markov chain Monte Carlo methods, in particular the No-U-Turn sampler, an adaptive form of Hamiltonian Monte Carlo sampling.[2,3]

As with compartmental models generally, additional assumptions of the model include a closed population and random mixing. The hospitalization model used in this analysis also made a number of simplifying assumptions, with the aim of decreasing the number of parameters to be estimated from data, an important consideration particularly early during the pandemic when reliable data available to calibrate the model were sparse. Specifically, the hospitalization model used here partitioned hospitalized patents into

© 2021 Geng EH et al. *JAMA Network Open.*

ICU and non-ICU patients, rather than explicitly modeling transitions between these states. It further made the simplifying assumption that all deaths occur among ICU patients. The current version of the LEMMA model (V 2.0) relaxes the above simplifying assumptions and uses a hospital model with more complexity. Due to the early stage of the epidemic to which it was applied, the version of the model used in this analysis further did not incorporate the possibility of waning immunity or re-infection. Version 2.0 allows for (user-specified) transitions from a recovered immune state back to a susceptible state.

In the current analysis, the model was calibrated to hospital census data only, given the sparsity of reliable testing data and other data sources available early during the pandemic. LEMMA supports the option to calibrate to multiple additional data sources, including deaths, cases, hospital admissions, ICU use, and, in Version 2.0, regional seroprevalence estimates and vaccine doses distributed. Incorporation of variants is also supported via input parameters on relative variant prevalence and growth rates, as well as epidemiologic characteristics of the variants (eg., relative transmissibility and severity, vaccine efficacy after first and second dose). Given the early stage of the epidemic to which it was applied, however, the current analysis employed a simpler model structure with no vaccine deployment and a single variant.

All code is open source and available on Github (https://github.com/LocalEpi/LEMMA/). Users can flexibly modify prior distributions on parameters, data inputs, and scenarios considered (including vaccine deployment, changes in effective contact rate, and variants) through either an Excel interface available on Github, or via an R shiny Application (https://localepi.shinyapps.io/LEMMA-Shiny; with automated data integration for all California counties).

References

1. Schwab, JS; Balzer L; Peng, J; Geng E; Petersen, M. Local Epidemiological Modeling for Management and Action . https://github.com/LocalEpi/LEMMA/; https://localepi.shinyapps.io/LEMMA-Shiny. Version 1.0. The MIT Open Source License (MIT) Copyright (c) 2020 LEMMA.
2. Stan Development Team. 2021. Stan Modeling Language Users Guide and Reference Manual, 2.27. https://mc-stan.org
3. Bob Carpenter, Andrew Gelman, Matthew D. Hoffman, Daniel Lee, Ben Goodrich, Michael Betancourt, Marcus Brubaker, Jiqiang Guo, Peter Li, and Allen Riddell. 2017. Stan: A probabilistic programming language. *Journal of Statistical Software* 76(1). 10.18637/jss.v076.i01

© 2021 Geng EH et al. *JAMA Network Open.*

eFigure 1. Date of Social Distancing Policies in St. Louis

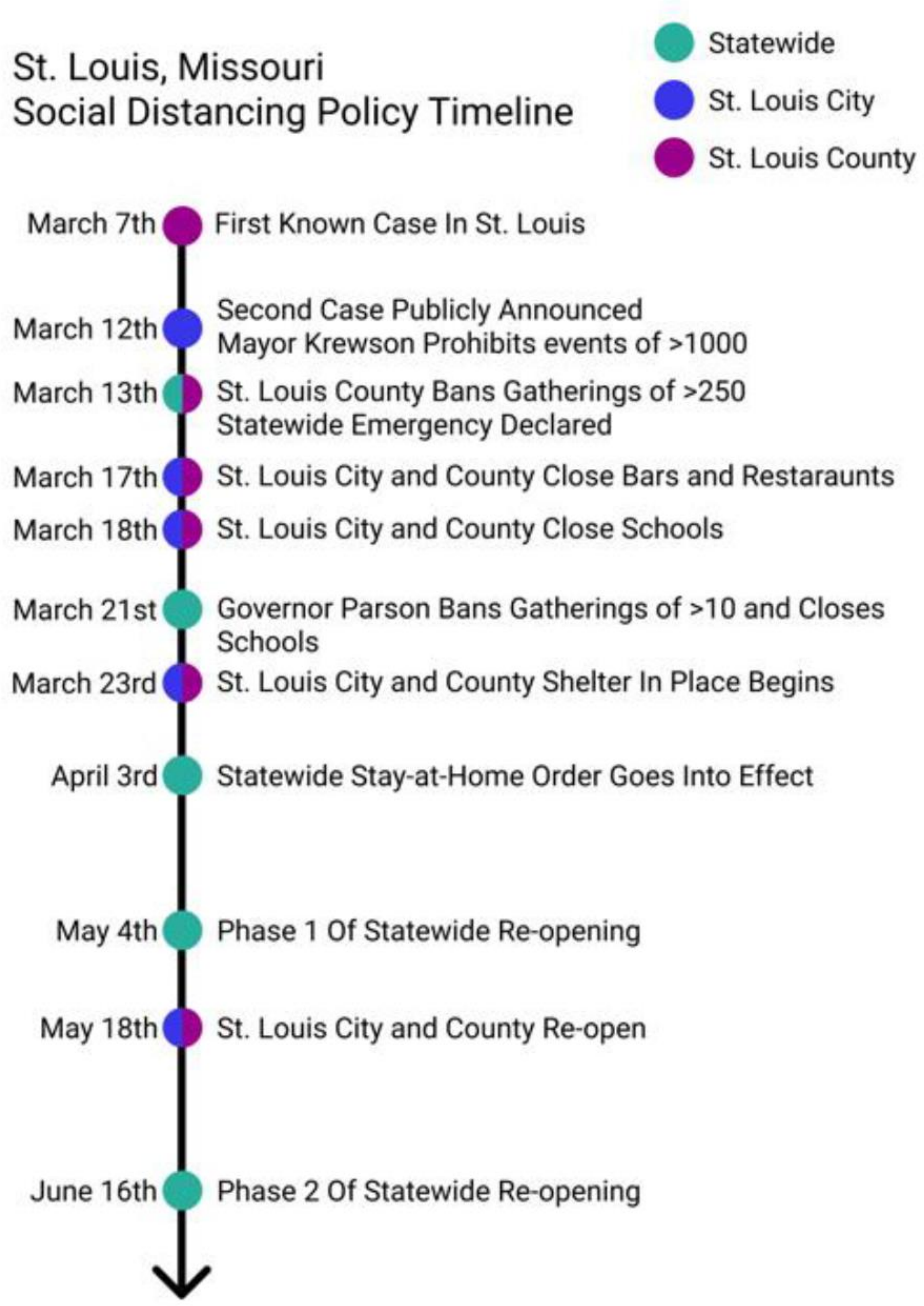

© 2021 Geng EH et al. *JAMA Network Open.*

eFigure 2. Projected Versus Actual COVID-19 Hospitalizations and Deaths

Using data from hospital census up until April 1 (Panel A) or May 1 (Panel B) — indicated as black vertical line on those dates — to calibrate the model, subsequent projections over time resemble actuals in both the number hospitalized over time as well as cumulative deaths. Projected and actuals were very similar over 12 weeks after last data input.

Panel A	Panel B

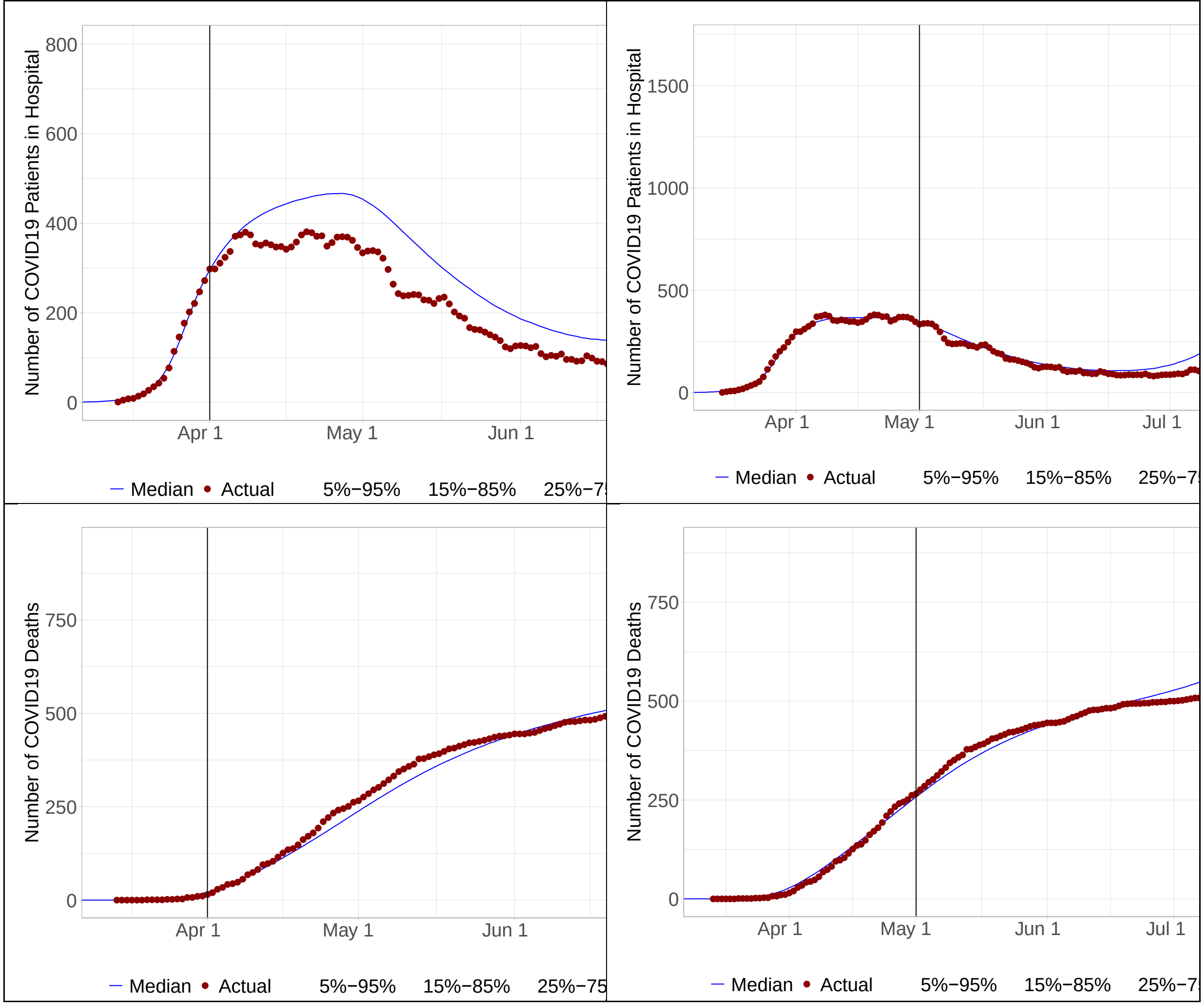

© 2021 Geng EH et al. *JAMA Network Open.*

eTable. Projected Hospitalizations and Deaths Under Additional Scenarios With Stronger Spontaneous Behavior Change

Projections exploring additional scenarios exploring counterfactual scenarios in which rapid behavior change occurs in the absence of social distancing policies and leads to an additional drop in effective reproductive number by 50% when hospitalizations reach a “panic” threshold of 600, 800 or 1000.

	**Actual number**	**Scenario 1: Policy intervention delayed by two weeks and replaced with 25% reduction in reproductive number due to spontaneous changes in behavior**	**Scenario 1 plus and additional drop in reproductive number due to spontaneous behavior change when hospitalizations reach 600**	**Scenario 1 plus and additional drop in reproductive number due to spontaneous behavior change when hospitalizations reach 800**	**Scenario 1 plus and additional drop in reproductive number due to spontaneous behavior change when hospitalizations reach 1000**
**Outcomes by April 15th**					
Hospital Census	348	2088 (1332 to 3260)	1858 (1271 to 2581)	1980 (1296 to 2781)	2026 (1333 to 2948)
Difference of Hospital Census	-	1740 (984 to 2912)	1510 (923 to 2233)	1632 (948 to 2433)	1678 (985 to 2600)
Percent Difference of Hospital Census	-	500 (282.8 to 836.8) %	433.9 (265.2 to 641.7) %	469 (272.4 to 699.1) %	482.2 (283 to 747.1) %
Cumulative Admits	1011	5221 (3425 to 7924)	4949 (3372 to 6918)	5132 (3391 to 7375)	5201 (3456 to 7540)
Difference of Cumulative Admits	-	4210 (2414 to 6913)	3938 (2361 to 5907)	4121 (2380 to 6364)	4190 (2445 to 6529)
Percent Difference of Cumulative Admits	-	416.4 (238.8 to 683.8) %	389.5 (233.5 to 584.3) %	407.6 (235.4 to 629.5) %	414.4 (241.8 to 645.8) %
Cumulative Deaths	115	327 (220 to 479)	320 (217 to 452)	326 (218 to 466)	325 (220 to 471)
Difference of Cumulative Deaths	-	212 (105 to 364)	205 (102 to 337)	211 (103 to 351)	210 (105 to 356)
Percent Difference of Cumulative Deaths	-	184.3 (91.3 to 316.5) %	178.3 (88.7 to 293) %	183.5 (89.6 to 305.2) %	182.6 (91.3 to 309.6) %
**Outcomes by May 15th**					
Hospital Census	221	768 (428 to 1333)	376 (242 to 565)	406 (265 to 621)	437 (295 to 662)
Difference of Hospital Census	-	547 (207 to 1112)	155 (21 to 344)	185 (44 to 400)	216 (74 to 441)
Percent Difference of Hospital Census	-	247.5 (93.7 to 503.2) %	70.1 (9.5 to 155.7) %	83.7 (19.9 to 181) %	97.7 (33.5 to 199.5) %
Cumulative Admits	1865	10427 (6511 to 15959)	7529 (5233 to 10383)	7980 (5396 to 11301)	8277 (5678 to 11842)
Difference of Cumulative Admits	-	8562 (4646 to 14094)	5664 (3368 to 8518)	6115 (3531 to 9436)	6412 (3813 to 9977)
Percent Difference of Cumulative Admits	-	459.1 (249.1 to 755.7) %	303.7 (180.6 to 456.7) %	327.9 (189.3 to 506) %	343.8 (204.5 to 535) %
Cumulative Deaths	384	1485 (946 to 2251)	1145 (799 to 1593)	1215 (827 to 1715)	1255 (856 to 1784)
Difference of Cumulative Deaths	-	1101 (562 to 1867)	761 (415 to 1209)	831 (443 to 1331)	871 (472 to 1400)
Percent Difference of Cumulative Deaths	-	286.7 (146.4 to 486.2) %	198.2 (108.1 to 314.8) %	216.4 (115.4 to 346.6) %	226.8 (122.9 to 364.6) %
**Outcomes by June 15th**					
Hospital Census	99	124 (60 to 257)	45 (28 to 73)	50 (31 to 81)	54 (35 to 88)
Difference of Hospital Census	-	25 (-39 to 158)	-54 (-71 to -26)	-49 (-68 to -18)	-45 (-64 to -11)
Percent Difference of Hospital Census	-	25.3 (-39.4 to 159.6) %	-54.5 (-71.7 to -26.3) %	-49.5 (-68.7 to -18.2) %	-45.5 (-64.6 to -11.1) %
Cumulative Admits	2246	11164 (6972 to 17204)	7633 (5291 to 10528)	8092 (5489 to 11487)	8414 (5811 to 11969)
Difference of Cumulative Admits	-	8918 (4726 to 14958)	5387 (3045 to 8282)	5846 (3243 to 9241)	6168 (3565 to 9723)
Percent Difference of Cumulative Admits	-	397.1 (210.4 to 666) %	239.8 (135.6 to 368.7) %	260.3 (144.4 to 411.4) %	274.6 (158.7 to 432.9) %
Cumulative Deaths	482	1904 (1181 to 2921)	1321 (921 to 1830)	1396 (952 to 1980)	1453 (1002 to 2070)
Difference of Cumulative Deaths	-	1422 (699 to 2439)	839 (439 to 1348)	914 (470 to 1498)	971 (520 to 1588)
Percent Difference of Cumulative Deaths	-	295 (145 to 506) %	174.1 (91.1 to 279.7) %	189.6 (97.5 to 310.8) %	201.5 (107.9 to 329.5) %
Peak Hospital Census and Date	381 (Apr 20)	2129 (Apr 18)	1860 (Apr 14)	1980 (Apr 15)	2026 (Apr 15)
Difference of Peak Hospital Census	-	1748	1479	1599	1645
Percent Difference of Peak Hospital Census	-	458.8%	388.2%	419.7%	431.8%

© 2021 Geng EH et al. *JAMA Network Open.*
